# Same-Day Discharge after Metabolic Bariatric Surgery: Methodological Heterogeneity, Discharge Success Rates and 30-Day Safety Outcomes — A Systematic Review

**DOI:** 10.1007/s11695-026-08880-2

**Published:** 2026-08-10

**Authors:** Mohamed Hany, Bart Torensma, Ahmed Ragab, Anwar Ashraf Abouelnasr, Daniel Bos, Arfan Ikram, Edo Aarts, Frits Berends

**Affiliations:** 1 Department of Surgery, Alexandria University, Alexandria, Egypt; 2 Madina Womens hospital, Alexandria, Egypt; 3 Department of Clinical Epidemiology, Erasmus MC, Rotterdam, Netherlands; 4 Department of Surgery, Weightworks Clinics, Amersfoort, Netherlands

**Keywords:** Same-day discharge, Metabolic bariatric surgery, Sleeve gastrectomy, Anastomotic procedures, Ambulatory surgery, Systematic review, Methodological heterogeneity, Safety outcomes

## Abstract

**Background:**

Same-day discharge after metabolic bariatric surgery (MBS) is increasingly practiced, but the validity of pooling published evidence remains unknown. Methodological heterogeneity in patient selection and discharge criteria may prevent valid meta-analysis. We systematically reviewed same-day discharge studies to assess whether criteria definitions are sufficiently standardized to permit evidence synthesis.

**Methods:**

We searched CENTRAL, PubMed, and EMBASE (inception to January 15, 2025) for studies reporting same-day discharge after MBS. We extracted inclusion criteria, exclusion criteria, and discharge criteria verbatim from each study and performed thematic content analysis to identify common components. Primary outcome was the proportion of studies using unique (non-shared) criteria definitions. Secondary outcomes included same-day discharge success rates and 30-day safety outcomes.

**Results:**

41 studies with 1,953,247 patients were included (4 RCTs, 23 cohorts, 11 registries, 3 other designs). Primary finding: Among 40 studies reporting inclusion criteria, 38 (95%) used unique definitions. Among 36 studies reporting exclusion criteria, 34 (94%) used unique definitions. Among 27 studies reporting discharge criteria, 27 (100%) used unique definitions. This heterogeneity was present across all study designs, including RCTs (100% unique criteria). Common components existed (age, BMI, ASA score) but thresholds varied dramatically. For example, age restrictions ranged from 16–69 years to 18–75 years across multiple different combinations. Secondary findings: Same-day discharge success rates ranged from 63 to 100% among studies reporting this outcome, but this variation primarily reflects discharge criteria stringency rather than true differences in feasibility or safety. Safety outcomes showed readmission rates of 0–20.8%, mortality of 0% in RCTs and cohort studies up to 0.12% in registries, and leak rates of 0–5.6%, but heterogeneous definitions prevented pooling. Stratified analysis revealed a differential safety signal, driven primarily by RYGB data, between sleeve gastrectomy and procedures involving an anastomosis.

**Conclusion:**

For the first time, this systematic review quantifies severe methodological heterogeneity in same-day discharge research (95–100% unique criteria definitions), violating fundamental assumptions for meta-analysis. Individual studies are well-conducted, but pooled estimates remain invalid when studies measure different interventions in different populations. Importantly, stratified analysis revealed a hypothesis-generating safety signal: same-day discharge after RYGB may carry higher mortality risk than after sleeve gastrectomy, reinforcing the need for procedure-specific standardization. International consensus on standardized patient selection criteria, discharge criteria definitions, and outcome reporting—separately for sleeve gastrectomy and anastomotic procedures—is urgently needed before additional studies are conducted.

**Supplementary Information:**

The online version contains supplementary material available at https://doi.org/10.1007/s11695-026-08880-2.

## Introduction

Traditionally, MBS required 1–3 days of postoperative hospitalization for monitoring, pain management, initiation of oral intake, and patient education. This approach was based on historical concerns about anastomotic complications, thromboembolic events, and the need for graduated nutritional advancement. However, enhanced recovery after surgery (ERAS) protocols [1] have fundamentally changed perioperative care across surgical specialties, including MBS.

Same-day discharge, defined as discharge home on postoperative day 0 (the day of surgery), represents the most intensive application of ERAS principles. Proponents argue that carefully selected patients can safely recover at home with appropriate discharge planning, patient education, and follow-up protocols. Potential benefits include reduced healthcare costs, decreased hospital-acquired infection risk, improved patient satisfaction, and increased operating room efficiency [1–3]. These advantages have motivated numerous centers worldwide to implement same-day discharge protocols.

Published same-day discharge studies report widely varying success rates (ranging from 15 to 100% in different series) and differing safety outcomes [4, 5]. As MBS surgeons, we need to understand whether these variations reflect true differences in safety and feasibility, or whether they represent methodological artifacts. This distinction has critical implications for clinical practice and guideline development.

From an epidemiological perspective, valid meta-analysis requires that studies measure the same intervention in comparable populations using similar outcome definitions. When patient selection criteria vary systematically across studies, the populations differ fundamentally in baseline risk. When discharge criteria definitions vary, the intervention itself differs—one study may be evaluating discharge of fully recovered patients while another evaluates discharge of partially recovered patients. When outcome definitions vary, studies are not measuring the same endpoints.

Traditional systematic reviews focus on pooling effect estimates to increase statistical power. However, this approach assumes studies differ only by random sampling error. When studies differ systematically in their populations, interventions, or outcome definitions, pooling reduces validity rather than increasing precision.

Previous systematic reviews have summarized safety outcomes of same-day discharge after MBS [4, 5], and most recently the IFSO Position Statement [6] presented a GRADE-based meta-analysis restricted to RCTs. However, no systematic review has quantified the methodological heterogeneity underlying this evidence base. While individual studies have been published, we lack understanding of whether these studies use comparable methodologies that would permit valid evidence synthesis. Without this knowledge, we cannot determine if published safety data can be pooled, generalized, or used to develop evidence-based guidelines.

The main endpoint of this systematic review was to assess methodological heterogeneity in patient selection criteria (inclusion and exclusion criteria) and discharge criteria across same-day discharge studies after MBS and determine whether this heterogeneity permits valid meta-analysis.

## Methods

This systematic review followed PRISMA 2020 guidelines [7] for reporting. The protocol was registered at PROSPERO (registration CRD42023487788), which pre-specified same-day discharge success rates as the primary outcome, with postoperative complications, readmission rates, and patient satisfaction as secondary outcomes. The protocol included systematic verbatim extraction of all inclusion criteria, exclusion criteria, and discharge criteria from each study. During this extraction process, we observed that the degree of methodological heterogeneity across studies was so extreme that it constituted the dominant finding, fundamentally affecting the interpretability of all registered outcomes. We therefore elevated methodological heterogeneity to the primary endpoint of the published review. This protocol deviation is reported transparently in accordance with PRISMA 2020 guidelines [7], and the originally registered outcomes are reported as secondary endpoints.

### Study Objectives

Primary objective: To assess methodological heterogeneity in patient selection criteria (inclusion and exclusion criteria) and discharge criteria across same-day discharge studies after MBS and determine whether this heterogeneity permits valid meta-analysis.

Secondary objectives: To summarize same-day discharge success rates and 30-day safety outcomes (complications, readmissions, mortality, reoperations) when reported.

### Eligibility Criteria

We included randomized controlled trials, cohort studies (prospective and retrospective), case–control studies, registry analyses, and cross-sectional studies examining same-day discharge after MBS in adults (age ≥ 18 years). Same-day discharge was defined as hospital discharge on postoperative day 0 (the calendar day of surgery). We included studies reporting any primary MBS procedure (sleeve gastrectomy, Roux-en-Y gastric bypass, adjustable gastric banding, biliopancreatic diversion, single-anastomosis duodeno-ileal bypass).

We excluded case reports, case series with fewer than 10 patients, review articles, editorials, commentaries, conference abstracts without full-text availability, and animal studies. We applied no language or date restrictions.

### Information Sources and Search Strategy

We conducted comprehensive searches: Cochrane Central Register of Controlled Trials (CENTRAL), PubMed/MEDLINE, and EMBASE. The search was conducted from database inception through January 15, 2025. We used a combination of MeSH terms and keywords related to: (1) bariatric or metabolic surgery (including specific procedure names), (2) same-day discharge or ambulatory surgery (including terms like outpatient, day surgery, day case), and (3) outcomes of interest. We also hand-searched reference lists of included studies and relevant review articles to identify additional studies not captured by electronic searches. Complete search strategies for each database are provided in Appendix Table S1.

### Study Selection

Two reviewers independently screened titles and abstracts. Afterwards, two reviewers independently assessed full-text articles for inclusion. Disagreements were resolved through discussion, with a third senior reviewer available for arbitration if consensus could not be reached.

### Data Collection Process

Three reviewers independently extracted data from the included studies. Extracted elements included: Study characteristics: First author, publication year, country, study design, study period, sample size (total population and same-day discharge group), surgical procedures included, follow-up duration, funding source.

Patient characteristics: Age, sex distribution, body mass index, American Society of Anesthesiologists (ASA) physical status classification, associated medical problems (diabetes, hypertension, sleep apnea, cardiovascular disease).

Methodological elements: Inclusion criteria (extracted verbatim), exclusion criteria (extracted verbatim), discharge criteria (extracted verbatim), discharge protocol components.

Outcomes: Same-day discharge success rate (number and percentage achieving discharge on day 0), complications (overall, major, minor), readmissions (number and percentage within 30 days), emergency department visits, reoperations, mortality (30-day), anastomotic leak or staple line leak rate.

For criteria extraction, we copied the exact text from each study without paraphrasing or interpretation. When studies did not explicitly state criteria, we extracted relevant text describing patient selection or discharge processes. This verbatim extraction was critical because subtle wording differences may reflect meaningful methodological distinctions.

### Risk of Bias Assessment

We assessed risk of bias using validated tools appropriate to study design. For randomized controlled trials, we used the Cochrane Risk of Bias tool version 2 (RoB 2) [8], which evaluates five domains: randomization process, deviations from intended interventions, missing outcome data, measurement of outcomes, and selection of reported results. Each domain was rated as low risk, some concerns, or high risk.

For cohort and case–control studies, we applied the Newcastle–Ottawa Scale, which assesses selection of cohorts, comparability of groups, and ascertainment of outcomes. For registry studies, we used an adapted quality assessment tool evaluating data source reliability, inclusion/exclusion criteria clarity, outcome definition, follow-up completeness, and adjustment for confounders.

### Statistics

Step 1: Verbatim criteria extraction: This text was compiled into a structured database organized by study design. We preserved the exact wording, punctuation, and formatting from the original publications to ensure we captured subtle differences in how criteria were defined.

Step 2: Unique definition identification: For each type of criteria (inclusion, exclusion, discharge), we compared the verbatim text across all studies to identify which studies used identical versus unique definitions. Two criteria sets were considered “identical” only if the verbatim text matched exactly or differed only in trivial formatting. Any difference in wording, thresholds, or component elements was classified as representing unique definitions. For example, “Age 18–60 years” and “Age 18–65 years” were classified as unique definitions despite both specifying age ranges.

Step 3: Component-level thematic analysis: We performed thematic content analysis to identify common components within each criteria type. For inclusion criteria, we identified recurring themes such as age restrictions, BMI criteria, ASA classification, primary surgery requirement, procedure type, distance from hospital, and social support requirements. For exclusion criteria, common themes included revision surgery, high-risk associated medical problems, anticoagulation therapy, psychiatric conditions, and substance abuse. For discharge criteria, common components included pain control thresholds, oral intake tolerance, vital sign stability, ambulation status, and time-based milestones.

Step 4: Keyword frequency analysis: For each identified component, we performed keyword-based frequency analysis across all studies. We developed a comprehensive list of keywords and synonyms for each component (e.g., for age: “age”, “years old”, “yo”). Using Python-based text analysis, we searched all criteria text for these keywords in a case-insensitive manner. We calculated the frequency with which each component appeared across studies, expressed as number of studies and percentage.

Step 5: Threshold variation analysis: For quantitative components (age ranges, BMI cutoffs, ASA scores, pain scale thresholds), they were analyzed for the distribution of these thresholds to quantify variation. For example, we identified all unique age ranges used across studies and counted how many different combinations existed.

Step 6: Heterogeneity quantification: We calculated the primary heterogeneity metric as: (Number of studies with unique criteria definitions/Total number of studies reporting criteria) × 100%. We calculated this separately for inclusion criteria, exclusion criteria, and discharge criteria. We also stratified this analysis by study design (RCTs, cohort studies, registry studies etc.) to determine whether heterogeneity varied by design quality.

Step 7: Interpretation threshold: We defined a priori that heterogeneity ≥ 80% (i.e., ≥ 80% of studies using unique definitions) would indicate that meta-analysis is inappropriate, as this suggests studies are measuring fundamentally different interventions or populations. This threshold is grounded in the Cochrane Handbook for Systematic Reviews of Interventions [9], which states that meta-analysis should only be considered when studies are “sufficiently homogeneous in terms of participants, interventions and outcomes to provide a meaningful summary,” and distinguishes clinical heterogeneity (variability in participants, interventions, and outcomes) from statistical heterogeneity (variability in effect estimates). The Handbook further emphasizes that clinical and methodological diversity always exist and that the decision to pool inevitably involves subjective judgment that must prioritize clinical comparability over statistical compatibility. Borenstein et al. [10] reinforce this principle, arguing that pooling across studies with fundamentally different populations or interventions produces a meaningless summary regardless of statistical homogeneity. Our ≥ 80% threshold operationalizes this principle: when at least four in five studies define the intervention, population, or outcome differently, the assumption of a common underlying effect is no longer tenable. Importantly, this threshold was conservative—our observed heterogeneity (95–100% unique definitions) far exceeded it.

### Data Synthesis and Meta-Analysis Approach

Meta-analysis was planned if methodological heterogeneity was acceptable (defined as < 20% of studies using unique criteria definitions). If criteria showed severe heterogeneity (≥ 80% unique definitions), meta-analysis would not be performed.

## Results

### Study Selection and Characteristics

**Fig. 1:**
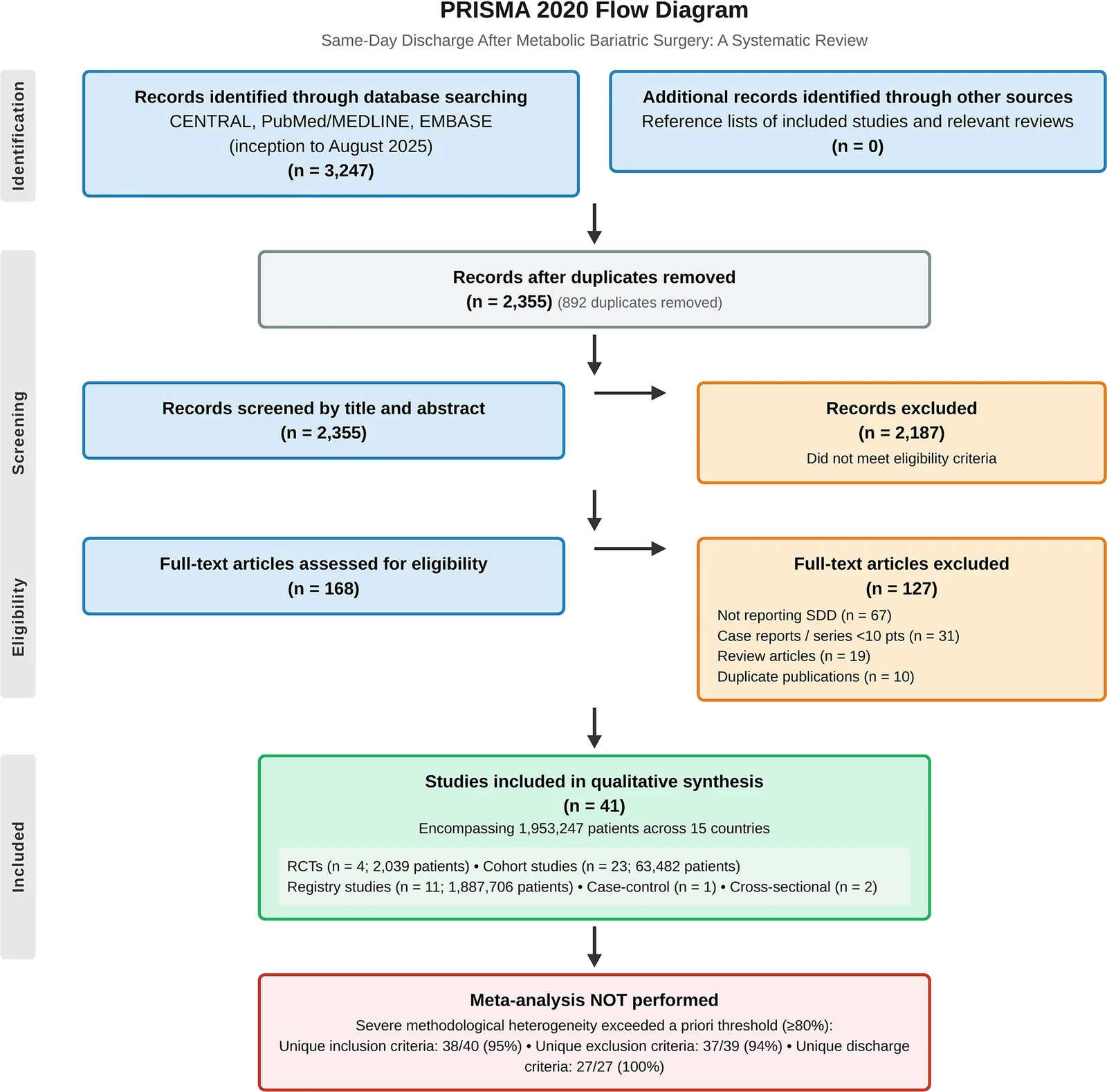
RISMA flow diagram

Our systematic search of CENTRAL, PubMed, and EMBASE databases identified 3,247 records. After removal of 892 duplicates, we screened 2,355 unique records by title and abstract, excluding 2,187 that did not meet eligibility criteria. We assessed 168 full-text articles for eligibility, ultimately including 41 studies that met all inclusion criteria [2, 3, 11–50]. The most common reasons for full-text exclusion were: not reporting same-day discharge (*n* = 67), case reports or small case series < 10 patients (*n* = 31), review articles (*n* = 19), and duplicate publications (*n* = 10) (Fig. 1).

The 41 included studies encompassed 1,953,247 patients across 15 countries. Study designs included 4 randomized controlled trials (2,039 patients), 23 cohort studies (63,482 patients), 11 registry studies (1,887,706 patients representing 96.7% of all patients), 1 case–control study, and 2 cross-sectional studies. Studies were published between 2005 and 2024, with 73% (30/41) published within the last 5 years, reflecting increasing interest in same-day discharge protocols.

Geographically, studies originated primarily from the United States (19 studies, 46%), Netherlands (3 studies), Canada (3 studies), and Saudi Arabia (2 studies), with additional studies from Norway, France, Spain, United Kingdom, Australia, and other countries. The large registry studies were predominantly from the United States utilizing the Metabolic and Bariatric Surgery Accreditation and Quality Improvement Program (MBSAQIP) database.

### Risk of Bias

Risk of bias assessment showed variable quality. Among the 4 RCTs, all demonstrated low risk of bias across all Cochrane RoB 2 domains. Among cohort studies, 12 (52%) were rated as low risk of bias using the Newcastle–Ottawa Scale, 10 (43%) as unclear risk, and 1 (4%) as high risk. Registry studies were predominantly rated as unclear risk due to inherent limitations in registry data quality and potential selection bias.

Complete study characteristics, including author, year, country, sample size, risk of bias ratings, and all outcome data, are presented in Table 1. Baseline patient characteristics and associated medical problems are detailed in Table 2.

**Table 1:** Characteristics and outcomes of all included studies

Author, Year	Country	Design	N	RoB	SDD Success n(%)	Leak n(%)	Mortality n(%)	Readmission n(%)	Reoperation n(%)
Aftab, 2019	Norway	RCT	93	Low	59 (63%)	1	0	3	1
Lodewijks, 2024	Netherlands	RCT	202	Low	65 (65%)	—	0 (0%)	4	—
Scheerhoorn, 2022	Netherlands	RCT	200	Low	NOT mentioned	—	—	—	—
Alqahtani, 2022	Saudi arabia	RCT	1544	Low	746 (96%)	0 (0%)	0 (0%)	1 (0.1%)	0 (0%)
Al-Masrouri, 2022	Canada	Cohort	320	Low	54 (96%)	0 (0%)	0 (0%)	1 (1.8%)	0 (0%)
Alshahrani, 2023	Saudi arabia	Cohort	53	Unclear	53 (100%)	1 (1.89%)	0 (0%)	1 (1.89%)	0 (0%)
Caballero, 2023	Spain	Cohort	14	Low	10 (71.5%)	0 (0%)	0 (0%)	0 (0%)	0 (0%)
Cooper, 2024	United states	Cohort	(1224) 940	Unclear	(940) (not mentioned%)	1 (0.1%)	—	18 (2.1%)	2 (0.2%)
Deffain, 2024	Canada	Cohort	72	Unclear	71 (98%)	0 (0%)	0 (0%)	5 (6.9%)	2 (2.8%)
Garofalo, 2015	Egypt	Cohort	328	Unclear	322 (98.2%)	2 (0.6%)	— (0%)	28 (8.5%)	1 (0.3%)
Katz-Summercorn, 2024	United kingdom	Cohort	288	Low	234 (81%)	—	—	17 (6%)	4 (1.4%)
Kleipool, 2023	Netherlands	Cohort	373	Low	373 (100%)	4 (1%)	0 (0%)	19 (5.1%)	5 (1.34%)
Kleipool, 2023	Netherlands	Cohort	49	Low	373 (100%)	4 (1%)	0 (0%)	19 (5.1%)	5 (1.34%)
Kleipool, 2023	Netherlands	Cohort	500	Low	373 (100%)	4 (1%)	0 (0%)	19 (5.1%)	5 (1.34%)
Kleipool, 2024	Netherlands	Cohort	50	Low	44 (88%)	0 (0%)	0 (0%)	1 (2%)	0 (0%)
Landreneau, 2024	United states	Cohort	75	Low	62 (82.7%)	—	—	0 (0%)	1 (1.3%)
Leepalao, 2019	United states	Cohort	362	Low	258 (71.5%)	3 (0.83%)	0 (0%)	13 (3.59%)	9 (2.49%)
McCarty, 2005	United states	Cohort	2000	Unclear	1669 (84%)	4 (0.5%)	2 (0.1%)	34 (1.7%)	—
Nijland, 2021	Netherlands	Cohort	50	Unclear	44 (88%)	—	—	2 (4%)	—
Rebibo, 2014	France	Cohort	100	High	92 (92%)	—	—	8	—
Surve, 2018	United states	Cohort	3162	Low	3154 (99%)	12 (0.4%)	0 (0%)	17 (0.6%)	18 (0.6%)
Thomas, 2016	Argentina	Cohort	2629	Unclear	2590 (98.2%)	26 (0.99%)	3 (0.11%)	4 (0.15%)	—
Van Ede, 2023	Netherlands	Cohort	102	Low	102 (100%)	—	—	7 (6.8%)	—
Fayed, 2018	Egypt	Cohort	96	Unclear	96 (not mentioned%)	1 (1.04%)	0	20 (20.8%)	2 (2.08%)
Morton, 2014	United states	Cohort	51788	Unclear	507 (not mentioned%)	—	4 (0.8%)	12 (2.4%)	—
Billing, 2013	United states	Cohort	250	Unclear	248 (99.2%)	1	0	9	1
Lalezari, 2018	United states	Cohort	821	Unclear	821 (100%)	7	0	17 (2.1%)	—
Schaffner, 2023	United states	Case control	20	Unclear	20 (100%)	0 (0%)	0 (0%)	2 (10%)	0 (0%)
Barbat, 2020	United states	Registry	408895	Unclear	9973	—	—	—	—
Aryaie, 2019	United states	Registry	271658	Unclear	7825	— (0.56%)	0	262 (3.35%)	65 (0.81%)
AbuHasan, 2024	United states	Registry	170127	Unclear	4203	—	—	—	—
Alam, 2024	United states	Registry	48408	Unclear	1918	—	3 (0.2%)	56 (2.9%)	20 (1%)
Mahan, 2024	United states	Registry	702622	Unclear	31308 (100%)	—	11 (0.03%)	654 (2.1%)	159 (0.5%)
Dreifuss, 2022	United states	Registry	167188	Unclear	2156 (100%)	—	1 (0.04%)	103 (4.8%)	30 (1.4%)
Dreifuss, 2022	United states	Registry	14624	Unclear	2156 (100%)	—	1 (0.04%)	103 (4.8%)	30 (1.4%)
Kleipool, 2024	Netherlands	Registry	9142	Unclear	755	5 (0.6%)	0 (0%)	300 (3.9%)	5 (0.6%)
Bharani, 2023	United states	Registry	Before matching 928,700. After matching 64,390	Unclear	After matching 33,848	—	38 (0.12%)	770 (2.39%)	210 (0.65%)
Inaba, 2018	United states	Registry	85,321	Unclear	4728	—	3 (0.94%)	— (3.45%)	— (1.88%)
Inaba, 2019	United states	Registry	9721	Unclear	319	—	4 (0.08%)	4 (2.14%)	29 (0.61%)
Rebibo, 2014	France	Cross sectional	100	—	—	—	—	8	—
Karam Ali, 2024	Canada	Cross sectional	265	—	—	0	0	4 (1.6%)	0 (0%)

*N* Number of patients; *RoB* Risk of bias; *SDD* Same-day discharge; *n(%)* Number and percentage; "—" Data not reported

**Table 2:** Baseline patient characteristics and associated medical problems

Author, Year	Design	N	DM	Hyperlipidemia	GERD	Hypertension	OSA	CPAP Use	Smoking	COPD	Hx DVT	Renal Insuff
Aftab, 2019	RCT	93	Yes	—	—	Yes	Yes	—	—	—	—	—
Lodewijks, 2024	RCT	202	Yes	—	—	Yes	—	—	—	—	—	—
Scheerhoorn, 2022	RCT	200	—	—	—	—	—	—	—	—	—	—
Alqahtani, 2022	RCT	1544	Yes	Yes	Yes	Yes	Yes	No	—	—	No	—
Al-Masrouri, 2022	Cohort	320	Yes	Yes	Yes	Yes	Yes	25.8%	—	—	—	—
Alshahrani, 2023	Cohort	53	Yes	Yes	No	Yes	No	No	—	—	—	—
Caballero, 2023	Cohort	14	Yes	Yes	Yes	Yes	—	—	—	—	—	—
Cooper, 2024	Cohort	(1224) 940	Yes	Yes	—	Yes	Yes	—	Yes	Yes	Yes	No
Deffain, 2024	Cohort	72	Yes	Yes	—	Yes	Yes	—	—	Yes	—	—
Garofalo, 2015	Cohort	328	Yes	Yes	No	Yes	Yes	—	—	—	—	—
Katz-Summercorn, 2024	Cohort	288	Yes	—	—	—	Yes	Yes	—	—	—	—
Kleipool, 2023	Cohort	373	Yes	Yes	—	Yes	Yes	28.7%	—	—	—	—
Kleipool, 2023	Cohort	49	Yes	Yes	—	Yes	Yes	28.7%	—	—	—	—
Kleipool, 2023	Cohort	500	Yes	Yes	—	Yes	Yes	28.7%	—	—	—	—
Kleipool, 2024	Cohort	50	Yes	Yes	—	Yes	Yes	—	Yes	—	—	—
Landreneau, 2024	Cohort	75	Yes	Yes	—	Yes	Yes	—	Yes	—	—	—
Leepalao, 2019	Cohort	362	Yes	Yes	Yes	Yes	Yes	—	—	No	—	—
McCarty, 2005	Cohort	2000	Yes	No	Yes	Yes	Yes	—	—	—	—	—
Nijland, 2021	Cohort	50	Yes	Yes	—	Yes	Yes	No	Yes	—	—	—
Rebibo, 2014	Cohort	100	Yes	Yes	—	Yes	No	—	—	—	—	—
Surve, 2018	Cohort	3162	Yes	Yes	Yes	Yes	Yes	—	—	—	—	—
Thomas, 2016	Cohort	2629	Yes	Yes	—	Yes	Yes	—	—	—	—	—
Van Ede, 2023	Cohort	102	Yes	—	Yes	Yes	Yes	—	—	—	—	—
Fayed, 2018	Cohort	96	Yes	Yes	—	Yes	Yes	—	—	—	—	No
Morton, 2014	Cohort	51788	—	—	—	—	—	—	—	—	—	—
Billing, 2013	Cohort	250	Yes	Yes	Yes	Yes	Yes	—	—	—	—	—
Lalezari, 2018	Cohort	821	—	—	—	—	—	—	—	—	—	No
Schaffner, 2023	Case control	20	Yes	Yes	Yes	Yes	Yes	—	—	—	—	—
Barbat, 2020	Registry	408895	Yes	Yes	Yes	Yes	Yes	—	Yes	Yes	Yes	Yes
Aryaie, 2019	Registry	271658	Yes	Yes	Yes	Yes	Yes	—	Yes	Yes	Yes	Yes
AbuHasan, 2024	Registry	170127	Yes	—	—	Yes	Yes	—	Yes	Yes	—	—
Alam, 2024	Registry	48408	Yes	Yes	Yes	Yes	Yes	—	Yes	Yes	Yes	Yes
Mahan, 2024	Registry	702622	Yes	Yes	Yes	Yes	Yes	—	Yes	Yes	Yes	Yes
Dreifuss, 2022	Registry	167188	Yes	Yes	Yes	Yes	Yes	—	Yes	—	Yes	Yes
Dreifuss, 2022	Registry	14624	Yes	Yes	Yes	Yes	Yes	—	Yes	—	Yes	Yes
Kleipool, 2024	Registry	9142	Yes	Yes	Yes	Yes	Yes	—	—	—	—	—
Bharani, 2023	Registry	Before matching 928,700. After matching 64,390	Yes	Yes	Yes	Yes	Yes	—	Yes	Yes	—	Yes
Inaba, 2018	Registry	85,321	Yes	Yes	—	Yes	Yes	—	Yes	Yes	Yes	Yes
Inaba, 2019	Registry	9721	Yes	Yes	Yes	Yes	Yes	—	Yes	Yes	Yes	Yes
Rebibo, 2014	Cross sectional	100	Yes	Yes	—	Yes	No	—	—	—	—	—
Karam Ali, 2024	Cross sectional	265	Yes	Yes	Yes	Yes	Yes	53%	—	—	—	—

*N* Number of patients; *DM* Diabetes mellitus; *GERD* Gastroesophageal reflux disease; *OSA* Obstructive sleep apnea; *CPAP* Continuous positive airway pressure; *COPD* Chronic obstructive pulmonary disease; *Hx DVT* History of deep vein thrombosis; *Renal Insuff.* Renal insufficiency; "—" Data not reported

### Inclusion Criteria

Among 40 studies that reported inclusion criteria, 38 (95%) used unique criteria not shared with any other study in the systematic review. Only 2 studies (both from the same research group) used identical inclusion criteria. This extreme heterogeneity was present across all study designs: RCTs (4/4, 100% unique), cohort studies (22/23, 96% unique), and registry studies (10/11, 91% unique). Notably, the 4 RCTs—which form the evidentiary basis for current clinical recommendations including the IFSO Position Statement [6]—each enrolled different populations: age ranges spanned from 18–50 to 18–65 years, BMI thresholds varied from 35–50 to 40–65 kg/m2, and ASA restrictions ranged from ASA 1–2 to ASA 1–3. These are not trivially different populations: a trial enrolling ASA 1–2 patients aged 18–50 with BMI 35–50 selects a low-risk cohort fundamentally different from one enrolling ASA 1–3 patients aged 18–65 with BMI 40–65. While common components existed across all studies, the specific thresholds and combinations varied.

Age criteria: 24 studies (59%) specified age restrictions, using multiple different age range combinations. Examples include: “18–50 years”, “18–60 years”, “18–65 years”, “18–70 years”, and “18–75 years”, with some studies using narrower ranges (e.g., “16–69 years”). The youngest upper limit was 50 years while the oldest was 75 years, representing a 25-year difference in patient age eligibility.

BMI criteria: 22 studies (54%) specified BMI restrictions, using multiple different cutoff combinations. The most restrictive study excluded patients with BMI > 45 kg/m2, while the most permissive accepted BMI up to 65 kg/m2. Common thresholds included BMI < 50, BMI < 55, and BMI < 60, but exact ranges varied (e.g., “BMI 35–50” vs “BMI ≥ 30” vs “BMI 35–60”).

ASA classification: 7 studies (17%) restricted eligibility by ASA physical status, with most requiring ASA 1–2 only while others accepted ASA 3. Restricting to ASA 1–2 selects a much healthier patient population than accepting ASA 3, creating systematic differences in baseline risk across studies.

Social support: 9 studies (22%) required some form of social support, ranging from “a caregiver available for 24 h” to “accompanied by a family member” to “responsible adult at home.”

Distance from hospital: 8 studies (20%) specified distance restrictions, ranging from “within 30 min” to “within 1 h driving distance” to “within 40 km radius.” Notably, each study defined the distance differently, adding yet another dimension of heterogeneity (Tables 3, 4, 5, 6, 7, 8, 9, 10, 11 and Appendix 1).

**Table 3:** Inclusion criteria components—RCTs (*n* = 4)

Inclusion component	Studies (n)	Percentage (%)	Notes
BMI criteria	1	25%	Aftab 2019
Willing to participate	2	50%	Lodewijks 2024, Scheerhoorn 2022
**Unique criteria**	**4**	**100%**	All 4 RCTs used different criteria

**Table 4:** Exclusion criteria components—RCTs (*n* = 4)

Exclusion component	Studies (n)	Percentage (%)	Notes
High ASA score	1	25%	Alqahtani 2022
Medical problems	2	50%	Aftab 2019, Alqahtani 2022
Psychiatric conditions	1	25%	Aftab 2019
Unique criteria	**3/3**	**100%**	All 3 RCTs with exclusion criteria used different criteria

Lodewijks 2024 did not report exclusion criteria

**Table 5:** Discharge criteria components—RCTs (*n* = 4)

Discharge component	Studies (n)	Percentage (%)	Notes
Pain control	3	75%	Aftab (VAS < 5), Lodewijks (oral meds), Scheerhoorn (oral meds)
Oral intake	1	25%	Aftab (oral water)
Vital signs stable	2	50%	Lodewijks (fever/HR/Hb), Alqahtani (within 20%)
Ambulation	2	50%	Aftab (without walking aid), Lodewijks (independent)
Nausea/vomiting	2	50%	Aftab (VAS ≤ 3), Lodewijks (controlled)
Urine output	1	25%	Aftab (elimination of urine)
Patient willing	1	25%	Lodewijks (willing to be discharged)
Hb/lab check	1	25%	Lodewijks (Hb decrease < 2 mmol/L)
Unique criteria	**4**	**100%**	All 4 RCTs used different discharge criteria

**Table 6:** Inclusion criteria components—Cohort studies (n = 23)

Inclusion component	Studies (n)	Percentage (%)
Age criteria	14	60.9%
BMI criteria	15	65.2%
ASA score	5	21.7%
Primary surgery	3	13.0%
LSG only	2	8.7%
Social support	6	26.1%
Distance from hospital	5	21.7%
Willing to participate	1	4.3%
Unique criteria	**22**	**96%**

**Table 7:** Exclusion criteria components—Cohort studies (*n* = 23)

Exclusion component	Studies (n)	Percentage (%)
Medical problems	11	47.8%
Age extremes	7	30.4%
Revision surgery	3	13.0%
High ASA score	3	13.0%
Anticoagulation	3	13.0%
Sleep apnea	2	8.7%
Distance	1	4.3%
Unique criteria	**18/20**	**90%**

3 cohort studies did not report exclusion criteria (Katz-Summercorn, Landreneau, van Ede). Garofalo 2015= Fayed 2018 (shared criteria)

**Table 8:** Discharge criteria components—Cohort studies (*n* = 23)

Discharge component	Studies (n)	Percentage (%)
Vital signs stable	13	56.5%
Pain control	9	39.1%
Oral intake	9	39.1%
Ambulation	9	39.1%
Nausea/vomiting	5	21.7%
Urine output	4	17.4%
Hb/lab check	5	21.7%
Patient willing	4	17.4%
No bleeding	4	17.4%
Time threshold	4	17.4%
Unique criteria	**23**	**100%**

4 cohort studies reported NA for discharge criteria (Leepalao, McCarty, Morton, Rebibo). Landreneau 2024 reported eligibility criteria rather than post-operative discharge criteria. Deffain 2024 referenced “modified PACU criteria” without specification

**Table 9:** Inclusion criteria components—Registry studies (*n* = 11)

Inclusion component	Studies (n)	Percentage (%)
Age criteria	6	54.5%
BMI criteria	3	27.3%
ASA score	1	9.1%
Primary surgery	3	27.3%
LSG only	3	27.3%
RYGB included	4	36.4%
Unique criteria	**10**	**91%**

**Table 10:** Exclusion criteria components—Registry studies (*n* = 11)

Exclusion component	Studies (n)	Percentage (%)
Revision surgery	7	63.6%
Age extremes	3	27.3%
High ASA score	3	27.3%
Unique criteria	**11**	**100%**

**Table 11:** Discharge criteria components—Registry studies (*n* = 11)

Discharge component	Studies (n)	Percentage (%)	Notes
No specific discharge criteria reported	11	100%	Registry studies defined SDD operationally as discharge on POD 0
Unique criteria	**11**	**100%**	All 11 registries used unique criteria

### Exclusion Criteria

Among 36 studies reporting exclusion criteria, 34 studies (94%) used unique exclusion criteria. Again, only 2 studies from the same group shared identical criteria. Heterogeneity persisted across study designs: RCTs (3/3, 100% unique), cohort studies (18/20, 90% unique), and registry studies (11/11, 100% unique).

Associated medical problems: 15 studies (42%) excluded patients with specific medical problems, but the definitions varied widely: “uncontrolled diabetes,” “insulin-dependent diabetes,” “severe cardiorespiratory disease,” “poorly controlled hypertension,” and “chronic kidney disease” were each defined differently or left unquantified.

Revision surgery: 11 studies (31%) excluded revision procedures, but 25 studies (69%) either included them or did not mention this exclusion.

Age extremes: 11 studies (31%) excluded patients at age extremes, with cutoffs ranging from “ > 45 years” to “ > 75 years” for upper limits.

High-risk ASA classification: 7 studies (19%) explicitly excluded high ASA scores, but with varying thresholds: some excluded ASA ≥ III, others ASA ≥ IV, and still others ASA class V or higher.

Obstructive sleep apnea: 4 studies (11%) excluded patients with sleep apnea, but with varying severity thresholds: some excluded any OSA, others only severe OSA requiring CPAP.

Anticoagulation therapy: 3 studies (8%) excluded patients on anticoagulation. Complete exclusion criteria component analysis is presented in Tables 3, 4, 5, 6, 7, 8, 9, 10, 11 and Appendix 2.

### Discharge Criteria

**Fig. 2:**
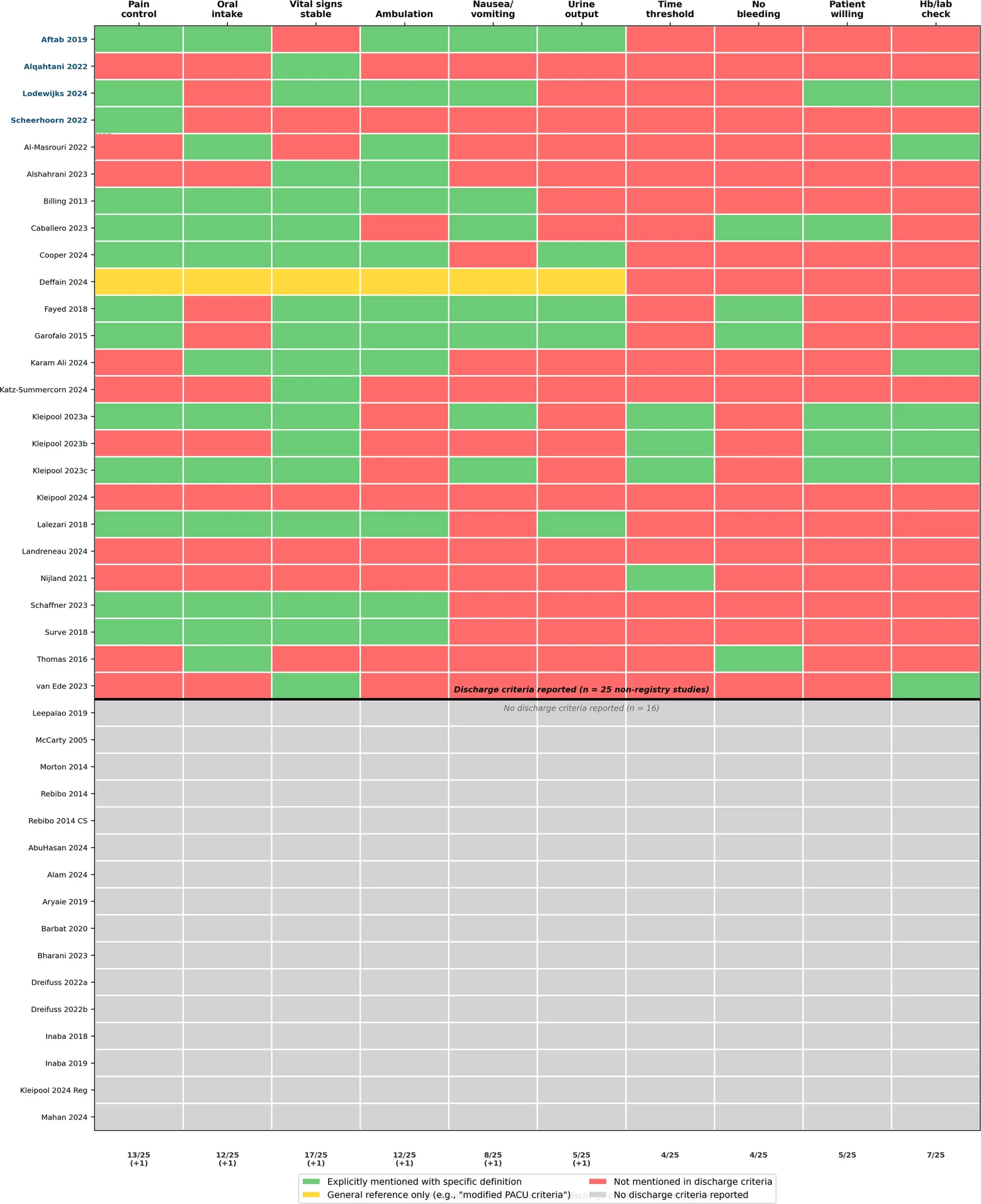
Heterogeneity heatmap

Among 27 studies reporting discharge criteria, all 27 studies (100%) used unique discharge criteria definitions. No two studies defined readiness for discharge identically, even when using similar component elements. This represents complete methodological heterogeneity—every single study defined the intervention differently (Fig. 2).

The variation in discharge criteria operates at two levels. First, most studies do not even report the same criteria domains. Second, when studies do mention the same domain, their specific definitions differ substantially:

Vital sign stability: 17 of 25 non-registry studies (68%) mentioned vital sign stability, but definitions ranged from unquantified (“hemodynamic stability”) to partially specified (“normal blood pressure and oxygen saturation”) to fully operationalized with specific thresholds (“HR < 90 bpm, SBP > 100 and < 150 mmHg, DBP > 60 and < 100 mmHg, temperature > 35.5 °C and < 37.5 °C, O2 saturation > 96%”). One study required “vital signs within 20% of baseline,” another required “heart rate < 100 bpm” as the sole vital sign criterion.

Pain control criteria: 13 of 25 non-registry studies (52%) explicitly mentioned pain control as a discharge criterion, but no two studies used the same definition. Quantified thresholds included VAS < 3, VAS < 5, and NRS > 4 (reverse-coded as absence of severe pain). Unquantified definitions included “pain well-controlled,” “optimal pain control,” “pain controlled with oral medication,” and simply “pain was controlled.”

Oral intake criteria: 12 of 25 non-registry studies (48%) required some oral intake tolerance, again with widely varying definitions. Specific volume requirements ranged from “30 ml of water every fifteen minutes” to “minimum oral intake of 200 ml of fluids postoperatively.” Others used unquantified descriptions: “tolerating liquids,” “clear liquids tolerance,” or “tolerating bariatric clear-liquid diet.”

Ambulation: 12 of 25 studies (48%) included ambulation, ranging from “normal mobility without the use of a walking aid” to “ambulating three times” to “ambulatory with normal room air saturation” to simply “ability to ambulate.”

Nausea/vomiting: 8 of 25 studies (32%) addressed nausea, from “nausea VAS ≤ 3” to “clinically important PONV” to “no nausea or vomiting” to “not too nauseous.”

Hb/lab check: 7 of 25 studies (28%) included a hemoglobin or laboratory criterion, with thresholds ranging from “hemoglobin decrease ≤ 1.0 mmol/L” to “ ≤ 2 mmol/L” to “ ≤ 3.2 g/dl” — different units and different cutoffs.

Urine output: 5 of 25 studies (20%) included urine output, from “elimination of urine and gas” to “voiding spontaneously” to “clear urine” to “able to void.”

Patient willingness: 5 of 25 studies (20%) explicitly required patient or surgeon approval for discharge.

Time threshold: 4 of 25 studies (16%) incorporated explicit time-based criteria, including “minimum 6 h of observation” and “operation before 11 o’clock.”

Notably, 14 of 41 studies (34%), predominantly large registry studies, did not report any specific discharge criteria, instead defining same-day discharge operationally as discharge on postoperative day 0 without specifying the clinical criteria used for discharge decisions. Complete discharge criteria component analysis across all study designs is presented in Tables 5 and 8, with the summary of heterogeneity findings across all criteria types in Table 12 and Appendix 3.

**Table 12:** Summary of methodological heterogeneity across all study designs

Study design	N studies	Unique inclusion (%)	Unique exclusion (%)	Unique discharge (%)	Poolable?
RCTs	4	100% (4/4)	100% (3/3)	100% (4/4)	NO
Cohort Studies	23	96% (22/23)	90% (18/20)	100% (23/23)	NO
Registry Studies	11	91% (10/11)	100% (11/11)	100% (11/11)	NO
Case–Control	1	—	—	—	N/A
Cross-Sectional	2	100% (2/2)	100% (2/2)	100% (2/2)	NO
Overall	**41**	**95% (38/40)**	**94% (34/36)**	**100% (27/27)**	**NO**

### Implications for Meta-Analysis Validity

The observed methodological heterogeneity (95% unique inclusion criteria, 94% unique exclusion criteria, 100% unique discharge criteria) far exceeds our a priori threshold (≥ 80%) indicating meta-analysis is inappropriate. Therefore, we did not perform meta-analysis (Table 12).

### Secondary Outcomes

Same-day discharge success rates (the proportion of patients initially selected for same-day discharge who successfully achieved discharge on day 0) were reported in 25 non-registry studies, ranging from 63 to 100% with a weighted mean of 94.0% (Table 1).

Critically, this variation reflects the heterogeneity in discharge criteria stringency rather than true differences in safety or feasibility. Studies using very strict criteria reported lower success rates: Aftab 2019 [11] (VAS < 3, tolerance of 100 mL liquid, specific vital sign ranges): 63% success. Lodewijks 2024 [12] (strict ERAS criteria): 65% success. Studies using more moderate criteria reported intermediate success: Caballero 2023 [13]: 71.5% success. Landreneau 2024 [14] (VAS < 5, tolerance of 50 mL): 83% success. Studies using lenient criteria or minimal quantification reported high success: Alqahtani 2022 [15] (vital signs within 20% of baseline, tolerance of liquids): 96% success. Surve 2018 [2] (patient feels ready, minimal specific criteria): 99% success. This gradient demonstrates that success rates are primarily a function of criteria stringency, not a reflection of superior protocols or patient selection.

Readmission rates: 30-day readmission rates were reported across 38 study entries (some studies reported multiple cohorts), ranging from 0% to 20.8% (Table 1 and Appendix 4). Representative examples from cohort studies: Kleipool 2023 [16] reported 5.1% readmission (19/373 patients). Al-Masrouri 2022 [17] reported 1.8% readmission (1/54 SDD patients). Fayed 2018 [18] reported the highest cohort readmission rate at 20.8% (20/96 patients). Thomas 2016 [19] reported 0.15% readmission (4/2,629 patients) in a large cohort. Among registry studies, rates ranged from 2.1% (Mahan 2024 [20]) to 4.8% (Dreifuss 2022 [21] LSG). The wide variation in readmission rates further illustrates the effect of heterogeneous patient selection and discharge criteria on outcomes.

**Fig. 3:**
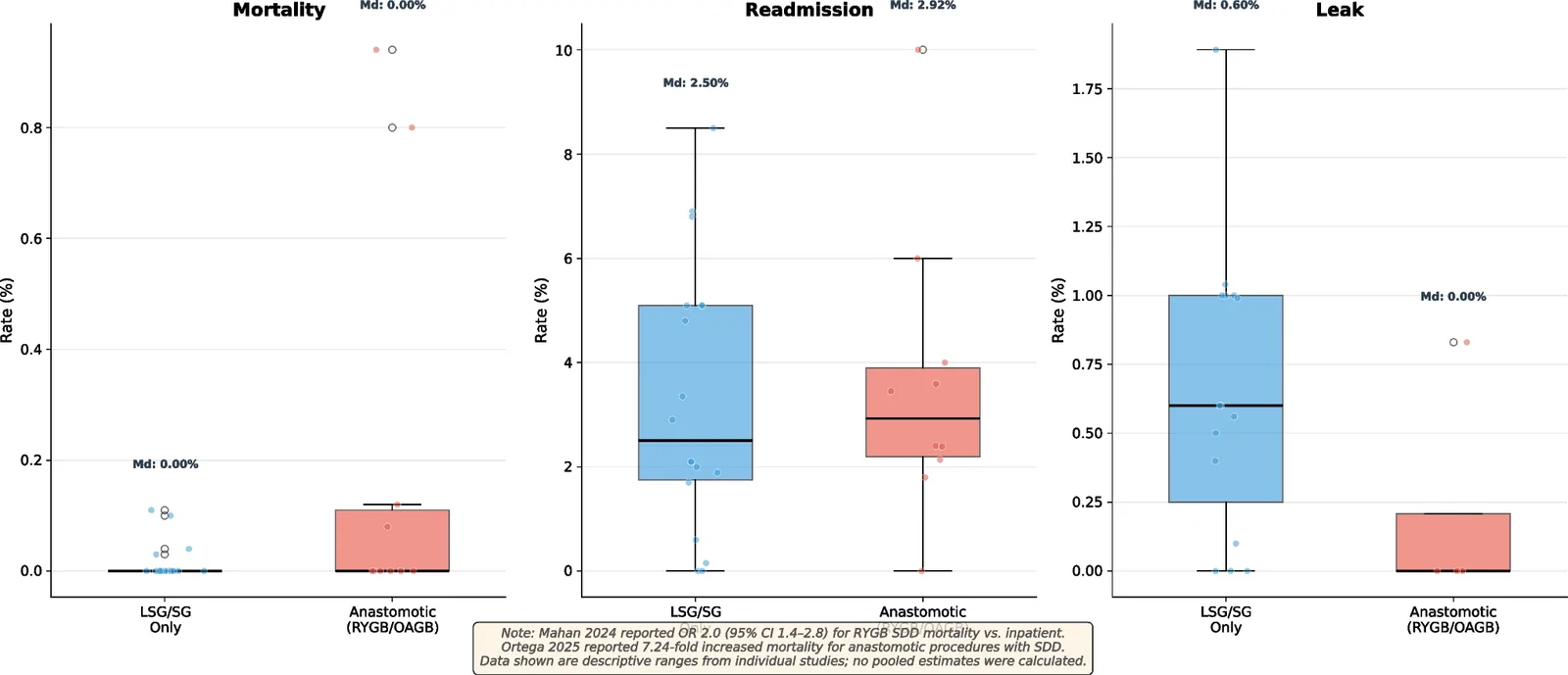
Safety signal

Mortality rates: 30-day mortality rates were reported across study designs. Among RCTs (2,039 patients), zero deaths occurred. Among cohort studies (63,482 patients), 9 deaths were reported: McCarty 2005 [22] (2 deaths, 0.1%), Thomas 2016 [19] (3 deaths, 0.11%), and Morton 2014 [23] (4 deaths, 0.8% of SDD patients). Among registry studies, 75 SDD-group deaths were reported across 9 studies: Mahan 2024 [20] (11 deaths, 0.03%), Dreifuss 2022 [24] RYGB (15 deaths, 0.1%), Bharani 2023 [25] (38 deaths, 0.12%), and others. Importantly, procedure-specific differences emerged. Mahan 2024 [20] (702,622 patients) found that LSG same-day discharge showed no increased mortality compared to standard care (OR 0.88, 95% CI 0.76–1.02, *p* = 0.09), whereas RYGB same-day discharge showed twofold increased mortality (OR 2.0, 95% CI 1.4–2.8, p < 0.001). This procedure-specific signal was further corroborated by Ortega et al. (2025) [4], whose meta-analysis—pooling four studies that were almost entirely RYGB-based—reported a 7.24-fold increased mortality risk for same-day discharge (OR 7.24, 95% CI 2.27–23.52, *p* = 0.001). Notably, the same meta-analysis found significantly fewer major complications with same-day discharge (OR 0.62, 95% CI 0.39–0.97, *p* = 0.04), indicating a more complex risk–benefit picture than the mortality signal alone suggests. These findings underscore that sleeve gastrectomy and RYGB have fundamentally different risk profiles in the context of same-day discharge (Fig. 3).

Leak rates: Anastomotic leak or staple line leak rates were reported in 24 study entries, ranging from 0% to 5.6% (Table 1 and Appendix 4). Among non-registry studies, rates ranged from 0% to 2.0% (Kleipool 2023 [16] RYGB cohort). The highest leak rate was reported by Dreifuss 2022 [24] for the RYGB registry subgroup (5.6%). Most leaks presented after the day of surgery (typically days 3–7). For example, Alshahrani 2023 [26] reported 1/53 patients (1.89%) developed leak that was diagnosed on day 5.

Reoperation rates: 30-day reoperation rates were reported in 29 study entries, ranging from 0% to 2.8% (Deffain 2024 [27]). Among non-registry cohort studies, other notable rates included Leepalao 2019 [28] (2.49%), Fayed 2018 [18] (2.08%), and Kleipool 2023 [16] RYGB (2.0%). Among registry studies, rates ranged from 0.5% (Mahan 2024 [20]) to 1.88% (Inaba 2018 [29]). The most common indications were bleeding (requiring return to OR for hemostasis), leak (requiring washout or revision), and early bowel obstruction.

## Discussion

This systematic review of 41 studies encompassing 1,953,247 patients reveals severe methodological heterogeneity in same-day discharge research after MBS. The primary finding was quantitative: 95% of studies use unique inclusion criteria, 94% use unique exclusion criteria, and 100% use unique discharge criteria definitions. This heterogeneity exists across all study designs, including high-quality randomized controlled trials.

Secondary findings showed same-day discharge success rates ranging from 63–100% (weighted mean 94.0%) and generally favorable short-term safety outcomes when reported, though with substantial variation. However, the methodological heterogeneity quantified in our primary analysis demonstrates that these outcomes cannot be validly pooled or compared across studies.

### Recognizing Individual Study Quality

It is essential to emphasize that our findings do not imply individual studies were poorly conducted or flawed. Many included studies represent rigorous, well-designed investigations that made important contributions to understanding same-day discharge feasibility in their specific populations and settings. The RCTs demonstrated low risk of bias across all quality domains. The large registry studies provided unprecedented statistical power. The prospective cohort studies offered detailed patient-level data with comprehensive follow-up.

Each study answered its specific research question within its chosen population and methodology. For example, a study demonstrating 96% same-day discharge success in highly selected ASA 1–2 patients aged 18–50 with strict discharge criteria provides valuable proof-of-concept that same-day discharge is achievable in ideal candidates. A registry study showing no mortality difference between same-day and standard discharge in LSG patients provides reassuring safety data for that specific procedure.

The problem is not individual study quality but collective heterogeneity. As a field, we have conducted 41 well-executed studies using 38 different inclusion criteria, 34 different exclusion criteria, and 27 different discharge criteria. Each study is internally valid, but they collectively fail to build a synthesizable evidence base. This represents a challenge for our surgical community: how do we move from conducting individual studies to building collaborative evidence that answers broader questions?

### The Epidemiological Problem: Why Heterogeneity Prevents Valid Synthesis

From an epidemiological perspective, when inclusion and exclusion criteria vary systematically across studies, the enrolled populations differ fundamentally in baseline characteristics and risk profiles. A study restricting enrollment to ASA 1–2 patients aged 18–50 with BMI 35–45 enrolls a low-risk population with excellent physiological reserve and minimal associated medical problem burden. In contrast, a study accepting ASA 3 patients aged 18–70 with BMI 35–65 enrolls a high-risk population with significant associated medical problem and diminished physiological reserve.

These populations have different baseline probabilities of complications, different recovery trajectories, and different tolerance for same-day discharge. Comparing outcomes between such heterogeneous populations conflates patient selection effects with intervention effects. We cannot determine whether observed differences in readmission rates, for example, reflect differences in discharge protocols or simply differences in the patients selected.

### Intervention Definition and Measurement Validity

When discharge criteria definitions vary, studies are measuring different interventions masquerading under the same name. Consider for example two studies both labeled “same-day discharge after LSG”:

Study A defines discharge as: VAS pain < 3, tolerance of 100 mL clear liquid, vital signs within 10% of baseline, independent ambulation, urine output > 30 mL/hour, no nausea, discharge by 8:00 PM.

Study B defines discharge as: VAS pain < 7, any oral intake without vomiting, “acceptable” vital signs, able to walk with assistance, patient agrees, discharge by 11:59 PM.

Study A selects a subset of fully recovered patients who meet strict physiological criteria. Study B includes partially recovered patients who meet minimal functional criteria. These are fundamentally different interventions applied to different subsets of the postoperative population. Their safety profiles are not comparable because they represent different points on the recovery spectrum.

### Outcome Interpretation and Artifact Recognition

The wide variation in reported same-day discharge success rates (63–100%) illustrates how heterogeneous methodology creates outcomes that cannot be directly compared. Higher success rates do not necessarily indicate superior ERAS pathways, better patient selection, or more effective perioperative protocols. Instead, they often reflect more lenient discharge criteria. A study reporting 95% success using lenient criteria (VAS < 7, any oral intake) and another reporting 65% success using strict criteria (VAS < 3, tolerance of specific volume) are not measuring the same outcome. The first is measuring “percentage of patients achieving minimal recovery,” while the second measures “percentage achieving full recovery.” Pooling these percentages produces a meaningless composite representing neither outcome.

### Procedure-Specific Safety Considerations

Stratified analysis of safety outcomes reveals an important signal that further underscores the need for procedure-specific standardization. Same-day discharge after laparoscopic sleeve gastrectomy (LSG) appears to carry a different risk profile than same-day discharge after RYGB. Mahan 2024 [20], the largest registry study (702,622 patients), found no increased 30-day mortality for LSG same-day discharge compared to inpatient care (OR 0.88, 95% CI 0.76–1.02), but a twofold increased 30-day mortality for RYGB same-day discharge (OR 2.0, 95% CI 1.4–2.8). Ortega et al. [4] reported a 7.24-fold increased 30-day mortality risk for same-day discharge in their meta-analysis (OR 7.24, 95% CI 2.27–23.52, *p* = 0.001). Although Ortega et al. framed their analysis as addressing anastomotic procedures broadly, the four pooled studies were almost entirely RYGB-based, with no SADI-S data and only a minor OAGB contribution within one mixed cohort. The 7.24 figure should therefore be understood as a RYGB-driven signal rather than one applicable to all anastomotic procedures. Importantly, the same meta-analysis found significantly fewer major complications with same-day discharge (OR 0.62, 95% CI 0.39–0.97, *p* = 0.04), indicating that the risk profile of same-day discharge after RYGB is more nuanced than the mortality signal alone suggests.

A methodological note on the 7.24 figure is warranted. The central thesis of this review is that pooled estimates in this field are undermined by extreme heterogeneity in populations, interventions, and outcome definitions. In citing the Ortega mortality estimate, we are conscious that this figure is itself a pooled estimate derived from four observational studies, two of which the source authors rated at serious risk of bias, with a wide confidence interval (2.27–23.52) and a registry-dominated dataset. Ortega et al. themselves characterize this finding as hypothesis-generating. We invoke this figure not as a robust pooled effect—which would be inconsistent with our thesis—but as a convergent signal: the direction of the mortality finding aligns independently in Mahan’s single-registry analysis (OR 2.0), in Ortega’s pooled estimate (OR 7.24), and in the biological plausibility of delayed anastomotic complications. It is the consistency of direction across independent analyses, rather than the precision of any single pooled estimate, that warrants clinical caution pending confirmatory prospective data.

These procedure-specific differences have biological plausibility. Anastomotic procedures carry inherent risks of anastomotic leak, internal hernia, and mesenteric ischemia—complications that may present insidiously in the first 24–72 h and require rapid surgical intervention. Sleeve gastrectomy, while not without risk (staple line leak rates of 0–5.6% in our review), involves a simpler surgical construct without intestinal anastomoses.

Descriptive comparison of study-level outcomes shows that among LSG-focused studies, reported mortality rates clustered near 0% (median 0%), while anastomotic procedure studies showed a wider range with a higher median (Fig. 3). Readmission rates showed similar but less pronounced divergence. These descriptive findings cannot be pooled for the same methodological reasons identified in our primary analysis, but they suggest that future standardization efforts should be procedure-specific from the outset. Same-day discharge criteria that are safe for LSG may not be appropriate for RYGB or other procedures involving an anastomosis, though the current evidence base for non-RYGB anastomotic procedures (OAGB, SADI-S) is too limited to draw conclusions.

### Why Traditional Meta-Analysis Fails here

Meta-analysis is predicated on the assumption that included studies differ only by random sampling error around a true underlying effect. Statistical techniques (random-effects models, heterogeneity statistics) account for this random variation. However, when studies differ systematically in their populations (selection criteria) and interventions (discharge definitions), they are not estimating the same true effect. No statistical method can overcome this fundamental epidemiological problem. The I2 statistic would indicate high statistical heterogeneity, but the more fundamental issue is clinical and methodological heterogeneity in what is being measured.

### Heterogeneity as External Validity: an Alternative Interpretation

It could be argued that consistently favorable outcomes across heterogeneous study populations constitute evidence of external validity for same-day discharge—if 41 studies with very different populations all report success rates of 63–100%, this demonstrates that same-day discharge works across a wide range of settings. However, this interpretation presupposes that the intervention is constant across studies—which our primary finding demonstrates it is not. External validity requires that the same intervention be tested in different populations; here, different interventions were applied to different populations using different outcome definitions. Even for hard endpoints such as mortality, the baseline risk profiles of enrolled patients differ systematically due to heterogeneous selection criteria, precluding valid cross-study comparison of event rates. A study enrolling only ASA 1–2 patients aged 18–50 with BMI < 45 has effectively removed the risk at the front door; its 0% mortality reflects study design, not necessarily intervention safety. A broader study enrolling ASA 1–3 patients up to age 70 with BMI up to 65 operates in a fundamentally different risk space. The zeros in these two studies are not equivalent—and that is confounding by indication at the study level.

### Relationship to the IFSO Position Statement

The recent IFSO GRADE-based Position Statement on same-day discharge after MBS [6] represents the most authoritative synthesis of this evidence to date. Kermansaravi et al. included 17 studies (46,578 patients) but deliberately restricted their primary meta-analysis to 4 RCTs (*n* = 2,026), finding no statistically significant differences between same-day discharge and inpatient care for complications (OR 1.49, 95% CI 0.63–3.52, *p* = 0.37), readmissions (OR 2.20, 95% CI 0.49–9.87, *p* = 0.30), or 30-day mortality (OR 0.87, 95% CI 0.12–6.22, *p* = 0.89).

Our findings do not contradict the IFSO conclusions but rather provide essential context for interpreting them. The IFSO group’s decision to restrict pooling to RCTs was methodologically sound precisely because it acknowledged the heterogeneity problem we quantify here—observational studies and registries were excluded from the primary analysis for well-documented methodological reasons. However, our data demonstrate that even within the RCT subgroup, the heterogeneity problem persists: all 4 RCTs (100%) used unique inclusion criteria, unique exclusion criteria, and unique discharge criteria definitions. The RCTs enrolled different patient populations (ASA 1–2 vs ASA 1–3, varying age and BMI ranges), applied different discharge criteria (strict physiological thresholds vs clinical judgment), and measured different outcome definitions. The IFSO meta-analysis pooled these 4 studies, producing non-significant results with notably wide confidence intervals—the readmission OR ranged from 0.49 to 9.87 and the mortality OR from 0.12 to 6.22—which may reflect not only small sample sizes but also the clinical heterogeneity between studies that we have identified.

Our work thus complements the IFSO Position Statement by providing the methodological audit that explains why the RCT evidence base yields imprecise estimates. The IFSO group concluded that same-day discharge is safe in appropriately selected patients—a conclusion we do not dispute at the individual study level. What our analysis adds is the demonstration that the field has not yet agreed on what “appropriately selected” means, with 95–100% of studies defining selection and discharge differently. Future iterations of IFSO guidelines would benefit from the standardized criteria we propose, which would enable more precise pooled estimates with narrower confidence intervals.

### Clinical Implications and Future Directions

Our findings have several immediate implications for clinical practice and research. First, clinicians should exercise caution when interpreting published success rates or safety outcomes, recognizing that these reflect specific—and heterogeneous—methodological choices rather than universally generalizable results. A “100% success rate” at one center using lenient criteria is not comparable to a “65% success rate” at another using strict physiological thresholds. The danger of averaging such figures is that it obscures meaningful clinical distinctions and may create false reassurance.

Second, the hypothesis-generating mortality signal for RYGB suggests that same-day discharge protocols should be procedure-stratified from the outset. Centers currently offering same-day discharge after RYGB should weigh the available data carefully, particularly the converging mortality signals from Mahan 2024 [20] and Ortega 2025 [4], and ensure that patient selection and postoperative monitoring reflect the higher-risk profile of procedures involving an anastomosis.

Third, centers implementing same-day discharge protocols should, until consensus criteria exist, adopt the most commonly reported components identified in our thematic analysis—vital sign stability (with defined parameters; reported by 68% of studies), pain control (quantified by validated scale; 52%), oral intake tolerance (with specified volume; 48%), ambulation (48%), and assessment for nausea/vomiting (32%)—and document their exact definitions to facilitate future comparison.

Fourth, our field does not need the 42nd study using yet another unique inclusion criteria set. The path forward requires international consensus on standardization. Collaborative efforts through societies such as ASMBS, IFSO, and EAES should aim to develop consensus-based standardized criteria through a formal process (e.g., Delphi methodology). These should specify standardized inclusion criteria, exclusion criteria, discharge criteria, and outcome reporting definitions—separately for sleeve gastrectomy and anastomotic procedures.

Fifth, pending such consensus, centers should maintain rigorous local quality improvement databases and participate in multicenter registries using shared definitions wherever possible. Only through methodological alignment can we move from fragmented individual studies to synthesizable collective evidence that advances patient care.

### Path Forward: from Fragmentation to Synthesis

Our field now has nearly 2 million patients studied across 41 trials. The barrier to evidence-based guidelines is not insufficient sample size or inadequate study designs. Publishing additional studies using unique criteria will not advance knowledge—it will simply add to the fragmented literature.

The path forward requires international consensus on standardization. We need collaborative international efforts (through societies like ASMBS, IFSO, European organizations) to develop consensus-based standardized criteria. These should specify:

Standardized inclusion criteria: Age range (suggest 18–65 years), BMI range (suggest 35–55 kg/m2), ASA classification (suggest ASA 1–2 for initial protocols), procedure type (suggest starting with primary LSG).

Standardized exclusion criteria: Revision procedures, ASA ≥ 3, severe cardiopulmonary disease, severe sleep apnea requiring CPAP, anticoagulation therapy, psychiatric contraindications, substance abuse, inadequate social support, distance > 1 h from emergency surgical care.

Standardized discharge criteria: VAS pain < 5 on oral analgesia, tolerance of 100 mL clear liquid without nausea, vital signs within 20% of baseline (heart rate 60–100 bpm, systolic BP 90–160 mmHg, SpO2 > 95% on room air), independent ambulation, urine output > 0.5 mL/kg/hour, minimum 4 h postoperative observation, patient and caregiver education completed, understanding of warning signs, follow-up appointment scheduled, emergency contact information provided.

Standardized outcome reporting: 30-day complications (major and minor per standardized definitions), 30-day readmissions, 30-day emergency department visits, 30-day reoperations, 30-day mortality, patient satisfaction (using validated instrument).

### Implementation Challenges and Pragmatic Solutions

We acknowledge that implementing standardized criteria faces practical challenges: institutional differences in resources, varying insurance and healthcare systems, cultural differences in postoperative care expectations, medicolegal environments, and legitimate local adaptations. However, core criteria can be standardized while allowing documented deviations. Centers choosing different criteria should explicitly document deviations and rationale, enabling subgroup analysis rather than fragmenting the evidence base.

### Limitations

Several limitations warrant consideration. First, the inability to perform meta-analysis, while itself a key finding, prevents generating pooled safety estimates. Consequently, we could not perform sensitivity analyses, meta-regressions, or formal assessment of publication bias through funnel plots or Egger’s test—all of which require a meta-analytic framework that was precluded by our primary finding. Second, not all studies reported all outcomes, leading to variable data availability. Third, publication bias warrants substantial discussion. Because no meta-analysis was conducted, formal statistical assessment of publication bias (funnel plots, Egger’s test, trim-and-fill) could not be performed. However, several features of the included literature suggest that publication bias is a particularly important threat to validity in this field. The majority of cohort studies originated from single centers with established same-day discharge programs, representing enthusiastic early adopters who have self-selected into this practice. Centers that attempted same-day discharge protocols and abandoned them due to safety concerns or logistical failures are unlikely to have published their experiences, creating survivorship bias—we see only the programs that persisted. The geographic concentration (46% from the United States, with heavy reliance on MBSAQIP registry data) further limits the representativeness of the evidence base. Additionally, same-day discharge offers demonstrable cost savings, which may create financial incentives for favorable reporting. The registry studies, while providing large sample sizes, are subject to self-selection: centers contributing to MBSAQIP that perform same-day discharge may differ systematically in resources, case volume, and expertise from non-contributing centers. Finally, we note that no study in our review was specifically designed to report negative outcomes of same-day discharge—all framed their investigation around feasibility, safety, or equivalence, creating a structural bias toward favorable conclusions. The true complication rate of same-day discharge across all settings, including those not represented in the published literature, may therefore be higher than what our included studies suggest. Fourth, the retrospective nature of most included studies introduces potential confounding and selection bias. Fifth, variable follow-up duration (most studies 30 days, some shorter, some longer) affects outcome ascertainment. Sixth, our keyword-based analysis may miss subtle criteria differences described using unexpected terminology. Seventh, we cannot assess unpublished protocols that were attempted but abandoned, potentially creating survivorship bias. Eighth, formal GRADE assessment of certainty of evidence was not performed for the primary endpoint, as GRADE presupposes pooled effect estimates which our primary finding demonstrates are inappropriate for this evidence base. For secondary safety outcomes, we provide a narrative assessment of certainty. For 30-day mortality, the evidence is of very low certainty: while zero deaths were reported in RCTs (2,039 patients), these trials were underpowered for mortality detection, and registry data (which provide the statistical power) carry high risk of confounding and selection bias. The differential mortality signal between LSG and RYGB (7.24-fold increase in the Ortega meta-analysis [4], itself a pooled estimate from four predominantly RYGB-based observational studies with wide confidence intervals) derives from data with potential unmeasured confounding, further reducing certainty. For 30-day readmission rates, the evidence is of low certainty: reported rates varied widely (0–20.8%), but this variation is inseparable from differences in patient selection and discharge criteria stringency across studies. For complications and leak rates, certainty is similarly low due to heterogeneous outcome definitions, incomplete reporting, and the predominantly observational and retrospective study designs. Future studies using standardized definitions would be the most direct path to generating higher-certainty evidence suitable for formal GRADE assessment. Ninth, as noted in the Methods, methodological heterogeneity was not the pre-registered primary outcome in PROSPERO; same-day discharge success rates were. The elevation of heterogeneity to the primary endpoint was a data-driven decision made during extraction when it became clear that the degree of methodological variation precluded meaningful interpretation of all pre-registered outcomes. We believe this deviation strengthened rather than weakened the review, as it addresses a more fundamental question (can these studies be compared at all?) before attempting to summarize their findings. All originally registered outcomes are reported as secondary endpoints. Tenth, our systematic search was conducted through January 15, 2025, and several relevant studies have been published subsequently, including Varban et al. [51], who reported higher rates of emergency department visits after same-day discharge sleeve gastrectomy (9.2% vs 6.2%, *p* = 0.003) in the Michigan Bariatric Surgery Collaborative, and the IFSO Position Statement [6] itself. We acknowledge this temporal gap. However, we note that additional studies published after our search cutoff are unlikely to alter our primary finding: each new study using its own unique criteria definitions would only increase the numerator and denominator of our heterogeneity metric proportionally, further reinforcing rather than undermining the conclusion that the field lacks standardization. The Varban et al. findings are consistent with our observation that outcome variation reflects methodological heterogeneity, and the IFSO Position Statement—while not identified in our original search—is now substantively discussed in this revised manuscript.

## Conclusion

Individual studies were well-conducted and answered their specific research questions. The barrier to evidence synthesis is not insufficient data but lack of methodological standardization: 95–100% of studies used unique criteria definitions, preventing valid meta-analysis. This heterogeneity persists even among the 4 RCTs that form the basis of the IFSO Position Statement [6], contributing to the wide confidence intervals observed in that analysis. Furthermore, a hypothesis-generating mortality signal for same-day discharge after RYGB—with converging evidence from independent registry and pooled analyses [4, 20]—underscores that one-size-fits-all criteria are inadequate and that procedure-specific risk profiles must inform future protocols.

The path forward requires collaborative efforts through international surgical societies (ASMBS, IFSO, EAES) to establish consensus-based standardized criteria—separately for sleeve gastrectomy and anastomotic procedures—covering patient selection, discharge definitions, and outcome reporting. Such standardization would strengthen the evidentiary foundation for future position statements and enable meta-analyses with the precision needed to guide clinical practice. Centers implementing same-day discharge should adopt standardized definitions when possible, document deviations when necessary, and maintain rigorous quality improvement databases. Only through methodological alignment can we move from fragmented individual studies to synthesizable collective evidence that advances patient care.

## Supplementary Information

Below is the link to the electronic supplementary material.

Supplementary file1 (DOCX 11 KB)

Supplementary file2 (DOCX 13 KB)

## Data Availability

No datasets were generated or analysed during the current study.

## Declarations

### Declarations of Generative AI Use

Generative AI tools (Claude, Anthropic) were used during the preparation of this manuscript to assist with spelling and grammar checks.
