# Supplementary material for: Same-Day Discharge after Metabolic Bariatric Surgery: Methodological Heterogeneity, Discharge Success Rates and 30-Day Safety Outcomes — A Systematic Review
Source: Obes Surg. 2026 Aug 10;36(9):4991–5010. doi: 10.1007/s11695-026-08880-2 (PMC13562099; doi:10.1007/s11695-026-08880-2)
Supplement: Supplementary file 1 [file 11695_2026_8880_MOESM1_ESM.docx]

# Appendix 1. Inclusion Criteria Components Across All Studies (n=41)

| Inclusion Criterion Component | Studies (n) | Percentage (%) | Notes |
| --- | --- | --- | --- |
| Age | 24 | 58.5% | 24/41 studies |
| BMI | 22 | 53.7% | 22/41 studies |
| Social support | 9 | 22.0% | 9/41 studies |
| Distance from hospital | 8 | 19.5% | 8/41 studies |
| ASA score | 7 | 17.1% | 7/41 studies |
| Primary surgery | 7 | 17.1% | 7/41 studies |
| LSG only/included | 7 | 17.1% | 7/41 studies |
| RYGB included | 7 | 17.1% | 7/41 studies |
| Willing to participate | 3 | 7.3% | 3/41 studies |
| **UNIQUE CRITERIA** | **38** | **95%** | 38/40 studies with criteria used unique inclusion criteria |

Note: 1 study (Cooper 2024) did not report inclusion criteria.

# Appendix 2. Exclusion Criteria Components Across All Studies (n=41)

| Exclusion Criterion Component | Studies (n) | Percentage (%) | Notes |
| --- | --- | --- | --- |
| Comorbidities | 15 | 36.6% | 15/41 studies |
| Revision surgery | 11 | 26.8% | 11/41 studies |
| Age extremes | 11 | 26.8% | 11/41 studies |
| High ASA score | 7 | 17.1% | 7/41 studies |
| Sleep apnea | 4 | 9.8% | 4/41 studies |
| Anticoagulation | 3 | 7.3% | 3/41 studies |
| Distance | 1 | 2.4% | 1/41 studies |
| Psychiatric conditions | 1 | 2.4% | 1/41 studies |
| **UNIQUE CRITERIA** | **34** | **94%** | 34/36 studies with criteria used unique exclusion criteria |

Note: 5 studies did not report exclusion criteria (Lodewijks 2024, Katz-Summercorn 2024, Landreneau 2024, van Ede 2023, Lalezari 2018).

# Appendix 3. Discharge Criteria Components Across Non-Registry Studies With DC Reported (n=25)

| Discharge Criterion Component | Studies (n) | Percentage (%) | Notes |
| --- | --- | --- | --- |
| Vital signs stable | 17 | 68.0% | 17/25 non-registry studies with DC |
| Pain control | 13 | 52.0% | 13/25 non-registry studies with DC |
| Oral intake | 12 | 48.0% | 12/25 non-registry studies with DC |
| Ambulation | 12 | 48.0% | 12/25 non-registry studies with DC |
| Nausea/vomiting | 8 | 32.0% | 8/25 non-registry studies with DC |
| Hb/lab check | 7 | 28.0% | 7/25 non-registry studies with DC |
| Urine output | 5 | 20.0% | 5/25 non-registry studies with DC |
| Patient willing | 5 | 20.0% | 5/25 non-registry studies with DC |
| No bleeding | 4 | 16.0% | 4/25 non-registry studies with DC |
| Time threshold | 4 | 16.0% | 4/25 non-registry studies with DC |
| **UNIQUE CRITERIA** | **27** | **100%** | All 27 studies with DC used unique discharge criteria definitions |

Note: 14 studies (predominantly registry studies) did not report specific discharge criteria. 2 studies with text in the discharge criteria column did not contain actual post-operative discharge criteria (Mahan 2024 reported demographics; AbuHasan 2024 reported recommendations). Denominator of 25 reflects non-registry studies that reported actual discharge criteria.

# Appendix 4. Same-Day Discharge Success Rates by Study Design

| Study Design | Studies (n) | Studies Reporting (%) | Success Rate Range | Weighted Mean |
| --- | --- | --- | --- | --- |
| RCT | 4 | 75% | 63.0% - 96.0% | 74.7% |
| Cohort | 23 | 87% | 71.5% - 100.0% | 91.6% |
| Registry | 11 | 27% | 100.0% - 100.0% | 100.0% |
| Case-control | 1 | 100% | 20.0% - 20.0% | 20.0% |
| Cross-sectional | 2 | 100% | 89.0% - 92.0% | 90.5% |
| **OVERALL** | **41** | **—** | **63% - 100%** | **88.5%** |
