# Supplementary material for: Same-Day Discharge after Metabolic Bariatric Surgery: Methodological Heterogeneity, Discharge Success Rates and 30-Day Safety Outcomes — A Systematic Review
Source: Obes Surg. 2026 Aug 10;36(9):4991–5010. doi: 10.1007/s11695-026-08880-2 (PMC13562099; doi:10.1007/s11695-026-08880-2)
Supplement: Supplementary file 2 [file 11695_2026_8880_MOESM2_ESM.docx]

PRISMA Search strategies for each database

**Database 1: PubMed/MEDLINE (inception to January 15, 2025)**

| Line | Search terms |
| --- | --- |
| #1 | “Bariatric Surgery”[MeSH] |
| #2 | “Gastric Bypass”[MeSH] |
| #3 | “Gastrectomy”[MeSH] |
| #4 | “Obesity, Morbid/surgery”[MeSH] |
| #5 | bariatric surger[tiab] OR metabolic surger[tiab] OR obesity surger*[tiab] |
| #6 | sleeve gastrectom*[tiab] OR gastric sleeve[tiab] OR LSG[tiab] |
| #7 | gastric bypass[tiab] OR Roux-en-Y[tiab] OR RYGB[tiab] OR LRYGB[tiab] |
| #8 | gastric banding[tiab] OR adjustable gastric band*[tiab] OR LAGB[tiab] |
| #9 | biliopancreatic diversion[tiab] OR duodenal switch[tiab] OR BPD-DS[tiab] |
| #10 | single anastomosis[tiab] OR one anastomosis[tiab] OR OAGB[tiab] OR SADI-S[tiab] |
| #11 | #1 OR #2 OR #3 OR #4 OR #5 OR #6 OR #7 OR #8 OR #9 OR #10 |
| #12 | “Ambulatory Surgical Procedures”[MeSH] |
| #13 | “Patient Discharge”[MeSH] |
| #14 | “Length of Stay”[MeSH] |
| #15 | same-day discharge[tiab] OR same day discharge[tiab] |
| #16 | outpatient[tiab] OR ambulatory[tiab] OR day-case[tiab] OR day case[tiab] |
| #17 | day surgery[tiab] OR day-surgery[tiab] OR daycase[tiab] |
| #18 | early discharge[tiab] OR short stay[tiab] OR short-stay[tiab] |
| #19 | 23-hour[tiab] OR 23-h[tiab] OR “23 hour”[tiab] OR “less than 24”[tiab] |
| #20 | outpatient surger[tiab] OR office-based surger[tiab] |
| #21 | ambulatory surger*[tiab] OR ambulatory care[tiab] |
| #22 | #12 OR #13 OR #14 OR #15 OR #16 OR #17 OR #18 OR #19 OR #20 OR #21 |
| #23 | #11 AND #22 |

Results: 1,482 records

**Database 2: EMBASE via Ovid (inception to January 15, 2025)**

| Line | Search terms |
| --- | --- |
| #1 | exp bariatric surgery/ |
| #2 | exp stomach bypass/ |
| #3 | exp sleeve gastrectomy/ |
| #4 | exp laparoscopic gastric bypass/ |
| #5 | (bariatric adj2 surger*).tw. |
| #6 | (metabolic adj2 surger*).tw. |
| #7 | (sleeve gastrectom* or gastric sleeve or LSG).tw. |
| #8 | (gastric bypass or Roux-en-Y or RYGB or LRYGB).tw. |
| #9 | (gastric band* or LAGB).tw. |
| #10 | (biliopancreatic diversion or duodenal switch or BPD-DS).tw. |
| #11 | (single anastomosis or one anastomosis or OAGB or SADI-S).tw. |
| #12 | 1 or 2 or 3 or 4 or 5 or 6 or 7 or 8 or 9 or 10 or 11 |
| #13 | exp ambulatory surgery/ |
| #14 | exp hospital discharge/ |
| #15 | exp length of stay/ |
| #16 | exp day surgery/ |
| #17 | (same-day discharge or same day discharge).tw. |
| #18 | (outpatient or ambulatory or day-case or day case).tw. |
| #19 | (day surgery or day-surgery or daycase).tw. |
| #20 | (early discharge or short stay or short-stay).tw. |
| #21 | (23-hour or 23-h or “23 hour” or “less than 24”).tw. |
| #22 | (ambulatory surger* or outpatient surger* or office-based surger*).tw. |
| #23 | 13 or 14 or 15 or 16 or 17 or 18 or 19 or 20 or 21 or 22 |
| #24 | 12 and 23 |
| #25 | limit 24 to (human and embase) |

Results: 1,203 records

**Database 3: Cochrane Central Register of Controlled Trials (CENTRAL) (inception to January 15, 2025)**

| Line | Search terms |
| --- | --- |
| #1 | MeSH descriptor: [Bariatric Surgery] explode all trees |
| #2 | MeSH descriptor: [Gastric Bypass] explode all trees |
| #3 | MeSH descriptor: [Gastrectomy] explode all trees |
| #4 | (bariatric surger* OR metabolic surger* OR obesity surger*):ti,ab,kw |
| #5 | (sleeve gastrectom* OR gastric sleeve OR LSG):ti,ab,kw |
| #6 | (gastric bypass OR Roux-en-Y OR RYGB OR LRYGB):ti,ab,kw |
| #7 | (gastric band* OR LAGB):ti,ab,kw |
| #8 | (biliopancreatic diversion OR duodenal switch OR BPD-DS):ti,ab,kw |
| #9 | (single anastomosis OR one anastomosis OR OAGB OR SADI-S):ti,ab,kw |
| #10 | #1 OR #2 OR #3 OR #4 OR #5 OR #6 OR #7 OR #8 OR #9 |
| #11 | MeSH descriptor: [Ambulatory Surgical Procedures] explode all trees |
| #12 | MeSH descriptor: [Patient Discharge] explode all trees |
| #13 | (same-day discharge OR same day discharge):ti,ab,kw |
| #14 | (outpatient OR ambulatory OR day-case OR day case):ti,ab,kw |
| #15 | (day surgery OR early discharge OR short stay):ti,ab,kw |
| #16 | (23-hour OR 23-h OR ambulatory surger* OR outpatient surger*):ti,ab,kw |
| #17 | #11 OR #12 OR #13 OR #14 OR #15 OR #16 |
| #18 | #10 AND #17 |

Results: 562 records

**Total records identified: 3,247** (PubMed: 1,482; EMBASE: 1,203; CENTRAL: 562). After removal of 892 duplicates, 2,355 unique records were screened.
